# TRPV4—A Missing Link Between Mechanosensation and Immunity

**DOI:** 10.3389/fimmu.2020.00413

**Published:** 2020-03-10

**Authors:** Laura Michalick, Wolfgang M. Kuebler

**Affiliations:** ^1^Institute of Physiology, Charité – Universitätsmedizin Berlin, Corporate Member of Freie Universität Berlin, Humboldt-Universität zu Berlin, and Berlin Institute of Health, Berlin, Germany; ^2^Institute of Physiology, Berlin Institute of Health, Berlin, Germany

**Keywords:** TRPV4, mechanosensation, innate immunity, infection, host defense, inflammation

## Abstract

Transient receptor potential vanilloid-type 4 (TRPV4) cation channel is widely expressed in all tissues as well as in immune cells and its function as mechanosensitive Ca^2+^ channel seems to be conserved throughout all mammalian species. Of late, emerging evidence has implicated TRPV4 in the activation and differentiation of innate immune cells, especially in neutrophils, monocytes, and macrophages. As such, TRPV4 has been shown to mediate neutrophil adhesion and chemotaxis, as well as production of reactive oxygen species in response to pro-inflammatory stimuli. In macrophages, TRPV4 mediates formation of both reactive oxygen and nitrogen species, and regulates phagocytosis, thus facilitating bacterial clearance and resolution of infection. Importantly, TRPV4 may present a missing link between mechanical forces and immune responses. This connection has been exemplary highlighted by the demonstrated role of TRPV4 in macrophage activation and subsequent induction of lung injury following mechanical overventilation. Mechanosensation via TRPV4 is also expected to activate innate immune cells and establish a pro-inflammatory loop in fibrotic diseases with increased deposition of extracellular matrix (ECM) and substrate stiffness. Likewise, TRPV4 may be activated by cell migration through the endothelium or the extracellular matrix, or even by circulating immune cells squeezing through the narrow passages of the pulmonary or systemic capillary bed, a process that has recently been linked to neutrophil priming and depriming. Here, we provide an overview over the emerging role of TRPV4 in innate immune responses and highlight two distinct modes for the activation of TRPV4 by either mechanical forces (“mechanoTRPV4”) or by pathogens (“immunoTRPV4”).

## Introduction

Mechanotransduction is a multistep process to convert mechanical stimuli into biochemical signals that elicit specific cellular effector functions. Over the past decade many key players in this mechanosensitive machinery have been identified, e.g., ion channels like transient receptor potential vanilloid-type 4 cation channel (TRPV4) (1, 2), PIEZO (3–5) and epithelial Na^+^ channel (ENaC) (6), cellular microcompartments such as primary cilia (7) or caveolae (8), or integrins which can sense the stiffness of the extracellular matrix (ECM) (9).

In injury, infection, or cancer immune cells are attracted by biochemical cues, yet during invasion of the affected tissue they encounter changes in the biophysical properties of the microenvironment, which in turn affect their functions. This emerging role of mechanical forces in immune responses has been termed mechanoimmunology (10). Moreover, in organs with a high dynamic mechanical load such as the lung or the heart immune cells face rapid changes in mechanical forces during the respiratory or cardiac cycle; yet relatively little is known about the effects of such mechanical cues on the innate immune system. During cell adhesion and migration immune cells are exposed to various biophysical stimuli including shear (in the circulation as well as during cell adhesion), deformation (when squeezing though narrow capillary passages or in transmigration), or cyclic mechanical stretch (in lung and heart as a result of ventilation or cardiac function). Although mechanical forces have been shown to impact signaling during adaptive immune processes (11–13), their effects on innate immunity are rarely considered and remain poorly understood. Recent studies, however, have implicated TRPV4 function in the regulation of innate immune responses (14–16). In the present review, we connect this emerging evidence with the established role of TRPV4 in mechanosensation to propose a novel concept of mechanoimmunology via TRPV4.

## MechanoTRPV4

The polymodal and non-selective TRPV4 cation channel, originally described by Strotmann et al. (17) and Liedtke et al. (18) in 2000, has been implicated over the past decades to act as a cellular mechanosensor in response to mechanical forces such as shear, stretch, osmotic swelling and shrinking, stiffening, and surface expansion (19–27) and is ubiquitously expressed in a wide range of cell types, including parenchymal cells such as smooth muscle cells, fibroblasts, epithelial cells, and endothelial cells as well as in immune cells, including macrophages, neutrophils (14, 16, 27–33).

TRPV4 activation mediates the influx of extracellular Ca^2+^, which can in turn activate Ca^2+^-triggered signaling cascades resulting in changes in transcription, vesicular transport, or cytoskeletal remodeling. The molecular mechanism how TRPV4 is activated by mechanical forces is currently not completely understood. At present, models for either direct or indirect mechanical activation of TRPV4 have been proposed (34, 35). The concept of direct activation is based on the assumption that an initial deformation of the plasma membrane's lipid bilayer will cause an expansion in cross sectional area, which creates a membrane tension-dependent energy difference followed by conformational change of the ion channel and thereby promotes force activation, as previously described for KCNK4 potassium channels by Brohawn et al. (36). This concept is supported by studies of Loukin et al. (37) who showed that TRPV4 can be activated by pipette suction in the presence of enzyme inhibitors in Xenopus oocytes, thus excluding mechanisms of indirect activation (37).

The concept of indirect TRPV4 activation follows the notion that TRPV4 is rather mechanically gated via intracellular signaling pathways such as integrin signaling, intracellular messengers and kinases, or simply by changes in surface expression (38, 39). Therefore, it has been demonstrated that forces applied to β_1_-integrins result in ultra-rapid activation of Ca^2+^ influx through TRPV4 channels and that the TRPV4 channels are rather activated by mechanical strain in the cytoskeletal backbone of the focal adhesion than by deformation of the lipid bilayer or peripheral cortical cytoskeleton (40). Such localized indirect activation is proposed to cause highly compartmentalized TRPV4-mediated Ca^2+^ signaling at focal adhesions and facilitates downstream activation of additional β_1_-integrins (integrin-to-integrin signaling) and leads to cell reorientation (40, 41). Moreover, several studies have shown that TRPV4 activation in response to osmotic or mechanical stress depends on formation of intracellular mechanomessengers, like lipid metabolites as arachidonic acid and its derivative 5′,6′-epoxyeicosatrienoic acid, and PIP_2_ (21, 42–45). Additionally, calmodulin as a classical second messenger binds to TRPV4 and mediates Ca^2+^ influx by conformational change and dissociation of its N- and C-terminus (20). It also has been observed that several protein kinases affect the activity of TRPV4 and/or facilitates binding to anchoring proteins (AKAPs) and F-actin and stabilize the channel in the plasma membrane (24, 27, 46–48). As such, a series of intracellular signaling cascades have been identified that modulate TRPV4 activity and may serve as pathways for indirect TRPV4 activation by mechanical forces.

In addition to the intracellular signaling pathways activating TRPV4, mechanical forces can affect TRPV4 trafficking and upregulate surface expression of the channel by recruitment from intracellular pools of TRPV4 to the plasma membrane via mechanoreceptive structures like calveolae, integrins, or adherens junctions (8, 26, 32, 41). Subcellular localization and trafficking of TRPV4 have been proposed to depend on pre- and post-translational modifications, like alternative splicing (49), nitrosylation, glycosylation and phosphorylation (27, 47, 50, 51). As these mechanisms were, however, largely identified using fluorescent-tagged overexpression systems their exact role in the trafficking of endogenous TRPV4 still remains incompletely understood.

**Figure 1:**
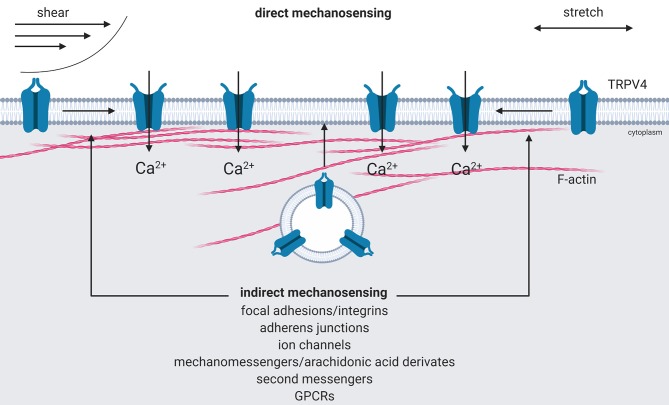
Activation of mechanoTRPV4. Direct activation of TRPV4 by shear or stretch forces results in an expansion in cross sectional area that creates a tension-dependent energy difference and leads to conformational changes of the channel by force activation (direct mechanosensing). Indirect activation is mediated by intracellular signaling cascades triggered via mechanosensitive focal adhesions or adherens junctions, ion channels, by intracellular mechano- or second messengers, G-protein-coupled receptors, e.g., protease-activated receptors that either activate TRPV4 or recruit it from intracellular pools to the plasma membrane (indirect mechanosensing). Created with BioRender.com.

In summary, TRPV4 may not only respond to diverse triggers via various modes (direct and indirect) of activation, diverse signaling pathways and protein modifications, but also different mechanisms to increase the abundance of open Ca^2+^ channels at the plasma membrane, respectively (Figure 1).

## ImmunoTRPV4

The role of TRPV4 in the innate immune system was first recognized more than a decade ago due to its thermosensitivity. Increments in body temperature in response to infection (i.e., fever) are important activators of the immune system with an evolutionary conserved role in host defense (52, 53). TRPV4 became first implicated in thermo-dependent immune modulation based on its role in thermal hyperalgesia (54–56). At the same time, similar effects were reported for other Ca^2+^ channels, such as the temperature-dependent activation of stromal interaction molecule (STIM) 1, which induces Ca^2+^ influx and in turn modulates gene expression and immune functions (57). These studies unveiled for the first time a direct link between Ca^2+^ channels, temperature, and immune function.

In 2008, Spinsanti and colleagues for the first time detected high expression levels of TRPV4 in human leukocytes (58). Subsequent functional studies from our group identified an important role for TRPV4 in regulating key neutrophil functions in response to pro-inflammatory stimuli like production of reactive oxygen species, cell adhesion, or migration (16). *In vivo, Trpv4*-deficient mice showed a marked protection from acute lung injury in two independent studies following either acid-induced or chlorine-induced lung injury (16, 59, 60). These effects were replicated by pharmacological inhibition of TRPV4, which similar to *Trpv4* deficiency attenuated characteristic signs of lung injury including hypoxemia, reduced compliance, edema formation, histological evidence of lung injury, and last not least, neutrophil infiltration and the release of pro-inflammatory cytokines (27, 59). Bone marrow chimeras from *Trpv4*-deficient and corresponding wild type mice revealed that the barrier protective effects in *Trpv4*-deficient mice was mostly attributable to a lack of TRPV4 in parenchymal tissue (presumably most relevant in endothelial cells), whereas TRPV4 deficiency in hematopoietic blood cells primarily reduced neutrophil infiltration into the injured lung (16). As such, it remains to be shown to which extent TRPV4-mediated activation of neutrophils affects organ function *in vivo*. In principle, these findings demonstrate that TRPV4 regulates neutrophil adhesion and migration, whereas in barrier forming cells like endothelial and epithelial cells, TRPV4 acts as a door opener for protein and fluid extravasation.

In macrophages, TRPV4 mediates both pro-inflammatory functions including phagocytosis, adhesion, and reactive oxygen species production, as well as anti-inflammatory effects and secretion of pro-resolution cytokines and bacterial clearance (14, 15). As such, macrophage TRPV4 may exert both protective and detrimental effects to the host tissue, by facilitating bacterial clearance in infection while promoting parenchymal injury in sterile inflammation (16, 27, 59). In a recent study, a similar double-edged role of mechanosennsation in the modulation of the innate immune response to sterile inflammation vs. bacterial infection was reported for another emerging mechanoimmunological cation channel, PIEZO1 (5).

An important additional role of TRPV4 in innate immunity relates to expression and function in the vascular endothelium, which by way of lining the inner surface of blood vessels and regulating cell adhesion and migration via expression of adhesion molecules acts as a gate keeper and controls the access for cells of the innate (and adaptive) immune system to sites of inflammation (61). TRPV4 activation in lung endothelial cells has been shown to increase vascular permeability (16, 27, 29), in part via disintegration of cell junctions (62) and degradation of ECM components and non-matrix components like integrins and VE-cadherins by matrix metalloproteinases (MMPs) MMP2 and MMP9 (63), and in part by calmodulin-dependent activation of the endothelial contractile machinery (64). As a consequence of these effects, TRPV4 activation results in endothelial detachment from the basal lamina, consecutive disruption of the endothelial barrier, and ultimately edema formation (65, 66). These effects seem to be most prominent in the pulmonary microvasculature (67), which may be related to TRPV4's roles in immunity and defense on the one, and the fact that the alveolo-capillary barrier presents a large surface for pathogen invasion on the other hand.

In lung alveolar epithelium, TRPV4 has been shown to act as a critical regular of epithelial barrier function, but at the same time revealed protective effects by increasing in bacterial clearance in large airways (31, 68, 69). All these findings identify TRPV4 as an important regulator of innate host defense responses including the regulation of phagocytes such as neutrophils and macrophages, but also of barrier forming epithelial and endothelial cells.

## TRPV4 in Host Defense

In line with its role in innate immune cells, TRPV4 has been implicated in different scenarios of host defense. In a murine model of *Streptococcus pneumoniae* infection TRPV4-deficiency prevented leukocyte infiltration, reduced bacterial load in the alveolar space, and attenuated characteristic features of lung injury (70). While these data unequivocally highlight the role of TRPV4 in host defense against gram-positive bacteria, the mechanism of TRPV4 activation by such bacteria or their pore-forming toxins such as pneumolysin (*S. pneumoniae*), α-hemolysin (*S. aureus*), or listeriolysin O (*L. monocytogenes*) is still unclear (70–72).

In gram-negative infections with bacteria such as *Escherichia coli* and *Pseudomonas aeruginosa*, TRPV4 activation by ECM stiffening during infection synergizes with LPS-stimulated TLR4 activation of p38 and thereby promotes host defense and resolution from lung injury (15, 73). Conversely, activation of protease-activated receptor (PAR)-2 by thrombin suppresses TRPV4 activity in macrophages and resolves lung injury (74). Similarly, PARs are also activated by neutrophil elastase (NE), matrix metalloproteases (MMPs) or other microbial proteases (33, 75–79) which has been implicated to degrade ECM and thereby causing remodeling and matrix stiffening during infection (33, 63, 73, 80).

Albeit the number of studies on TRPV4 in immune cells is still limited, TRPV4 emerges as a regulator of innate immunity and host defense and may sense mechanical changes of the extracellular environment during inflammation. Therefore, occurring mechanical forces are crucial for TRPV4-mediated immune response and regulate both pro- and anti-inflammatory effects, which may have both beneficial effects in terms of bacterial clearance and resolution from injury.

## TRPV4 in Mechanosensation of Immune Cells

Mechanical forces generated by hemodynamic forces or a factor of ECM composition under physiological and pathophysiological conditions are acting on immune cells and can be subclassified in (i) mechanical stretch by shape changes during cell passage through narrow capillary segments, (ii) shear stress acting on circulating or adherent immune cells as a function of blood flow or viscosity, and (iii) changes in substrate stiffness of the ECM induced by inflammation (81, 82).

In particular, mechanical stretch has been implicated as a central component in pathological processes at the alveolo-capillary barrier of the lung where it has been extensively studied in the context of ventilator-induced lung injury (VILI). In endothelial and epithelial cells, TRPV4 has been shown to become activated during mechanical stretch as exerted by mechanical (over-) ventilation leading to Ca^2+^ influx and subsequent loss of lung barrier integrity and the release of cytokines (27, 29, 68). For macrophages, TRPV4 function has been shown to be critical in the pro-inflammatory response to mechanical ventilation (14). As shown by Hamanaka and colleagues in studies on isolated-perfused mouse lungs, replacement of wild type with *Trpv4*-deficient macrophages in wild type lungs was sufficient to attenuate classical features of VILI, a finding that was linked to stretch-induced and TRPV4-dependent intracellular Ca^2+^ signaling, and the subsequent formation of reactive oxygen and nitrogen species *in vitro*.

In neutrophils, transmigration during VILI has so far largely been considered as a response secondary to the mechanical stretch on parenchymal cells (16, 83). In contrast to the systemic circulation where neutrophil adhesion and migration are primarily localized to postcapillary venules and mediated by adhesion molecules (61), the initial mechanism of neutrophil sequestration in the lung is largely based on cytoskeletal rearrangement and formation of F-actin rims which increase cellular stiffness and as a result, decrease their ability to change their shape from spherical to elliptical. These changes in deformability prevents activated neutrophils to pass through the narrow capillary segments of the alveolo-capillary network where they get trapped at sites of inflammation (84). While neutrophil stiffening was originally considered an irreversible feature, recent studies suggest that alternating neutrophil stiffening and softening can drive the dynamic oscillation of neutrophils between the activated/primed and deactivated/deprimed state (85). Since F-actin has been shown to bind to activated TRPV4, it can be speculated that this priming/depriming occurs as a function of TRPV4 activation secondary to the formation of F-actin rims (24). Given the implications of such mechanical effects due to e.g., changes in neutrophil shape and stiffness not only on neutrophil kinetics through the vascular system but also on their biological responsiveness in health and disease (86), the molecular dissection of the underlying signaling pathways and the potential link to TRPV4 mechanosensation may be of considerable scientific interest and relevance.

Shear stress in the vasculature is a result of blood flow velocity, vessel diameter, and blood viscosity and primarily acts on endothelial cells outlining the vessel lumen. Endothelial cells respond to shear stress by segregation of TRPV4 channels from β-catenin following relocating TRPV4 from adherens junctions to focal adhesions of the basal membrane which in turn increases endothelial permeability by destabilization of junctional complexes and Ca^2+^-mediated cytoskeletal remodeling (87). While it is reasonable to expect that circulating immune cells tethering or adhering to the vascular wall experience likewise considerable degrees of vascular shear stress, the effects of fluid shear stress on innate immune cells have so far not been extensively addressed. In the alveolar compartment, alveolar macrophages have been shown to contribute to VILI by secretion of pro-inflammatory mediators in a TRPV4-dependent manner (14). This effect is presumably predominantly caused by stretch rather than shear effects. Nevertheless, it is conceivable that shear-dependent activation of macrophages may become relevant in conditions of alveolar fluid accumulation when fluid will cyclically shift in and out of the alveolus resulting in considerable shear forces exerted not only on alveolar epithelial cells but also on alveolar macrophages (88, 89).

Finally, substrate stiffness is regulated by the composition of the ECM which changes as a function of physiological (development, aging) and pathophysiological (atherosclerosis, hypertension, fibrosis) processes (90–93). TRPV4 has been identified as a major mechanosensor for substrate stiffness, but so far this function has been exclusively attributed to parenchymal cells (30, 31, 94–96). Yet, it is fair to speculate that changes in substrate stiffness will similarly affect the mechanical forces that act upon immune cells during the processes of adhesion and transmigration, which accordingly may affect TRPV4-dependent cellular responses. Conversely, TRPV4-mediated activation of immune cells may in turn affect local ECM structure and composition by secretion of MMPs. As such, TRPV4 may play an important role in proteolytic disruption of ECM, cell-cell, and cell-matrix interaction by MMPs that is required for effective immune cell extravasation to sites of injury and inflammation (63). By similar mechanisms, TRPV4 may also contribute to chronic parenchymal remodeling, explaining its prominent role in tissue fibrosis in a positive feedback of substrate stiffening and TRPV4-mediated pro-fibrotic effects (30, 97).

In line with a critical role of substrate stiffness for immune cell function, the Ca^2+^ response to LPS in of macrophages were shown to correlate with substrate stiffness. Notably, such substrate stiffness-dependent modulation of macrophage signaling can alter macrophage phenotype toward an anti-inflammatory phenotype (M2) initiating bacteria clearance and resolution of lung injury (15, 73).

## Summary and Conclusion

This review provides an update on the role of TRPV4 in mechanosensation (“mechanoTRPV4”) on the one and inflammation and host defense (“immunoTRPV4”) on the other hand with the aim to point toward a possible, albeit still speculative, role of TRPV4 in mechanoimmunology.

**Figure 2:**
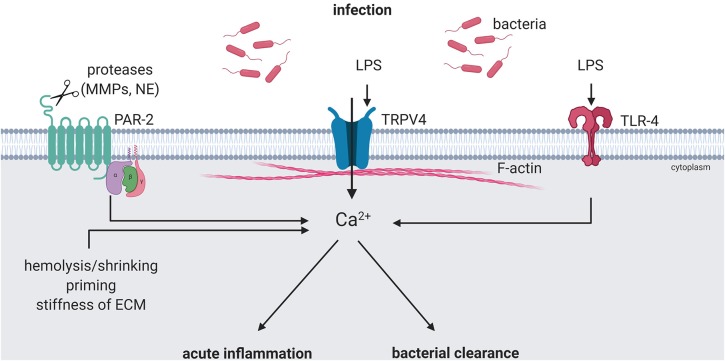
ImmunoTRPV4 activation during infection. Pathogen-associated signals, e.g., lipopolysaccharide (LPS) can modulate TRPV4 function directly or indirectly. Direct activation can lead to pro-inflammatory phenotypes and boost the response in acute inflammation. Activation of TLR-4 by LPS and cleavage of PAR-2 by release matrix metalloproteases (MMPs) and/or neutrophil elastase (NE) during inflammation can modulate the TRPV4-mediated immune response toward an anti-inflammatory phenotype and induce bacterial clearance. Created with BioRender.com.

Activation of mechanoTRPV4 directly impacts host defense in that it reduces endothelial and epithelial barrier function, but at the same time promotes the infiltration of innate immune cells and the release of pro- and anti-inflammatory cytokines (15, 16, 27). In addition, TRPV4 activation can regulate the delivery of circulating immune cells to local sites of inflammation and infection by mediating vasodilation (98, 99). Activation of immunoTRPV4 triggers and promotes inflammation and has emerged as a key regulator of bacterial clearance. Importantly, mechanosensitive and immunoregulatory functions of TRPV4 may not be distinct, but intrinsically linked, thus opening a new view on mechanoregulation of immune responses. This concept has already been well-established for epithelial and endothelial cells, where mechanical activation of TRPV4 has emerged a major regulator of barrier function and inflammatory responses. Yet, this notion has also been demonstrated for innate immune cells such as macrophages where Ca^2+^ signaling as a function of TRPV4-mediated sensing of substrate stiffness has been shown to shift the immune response from pro-inflammatory to an anti-inflammatory and resolving phenotype (15, 73) (Figure 2). Similar scenarios of mechanoregulation of immune cell function via TRPV4 may relate to a variety of scenarios where immune cells undergo changes in mechanical stress, such as during cell adhesion (shear stress) and transmigration (substrate stiffness, shape change), capillary transit (shape change) as well as tissue strain e.g., in mechanically ventilated lungs (stretch). So far, the link between mechanoTRPV4 and immunoTRPV4 has not been characterized in detail, but may provide for important insights into the regulation of innate immunity and host defense and as such, for the development of novel preventive, therapeutic, or adjuvant strategies in inflammatory and infectious diseases such as sepsis, pneumonia, or sterile inflammation as in VILI.

## Author Contributions

All authors listed have made a substantial, direct and intellectual contribution to the work, and approved it for publication.

### Conflict of Interest

The authors declare that the research was conducted in the absence of any commercial or financial relationships that could be construed as a potential conflict of interest.
